# High Field *In vivo* ^13^C Magnetic Resonance Spectroscopy of Brain by Random Radiofrequency Heteronuclear Decoupling and Data Undersampling

**DOI:** 10.3389/fphy.2017.00026

**Published:** 2017-07-28

**Authors:** Ningzhi Li, Shizhe Li, Jun Shen

**Affiliations:** Molecular Imaging Branch, National Institute of Mental Health, National Institutes of Health, Bethesda, MD, United States

**Keywords:** proton decoupling, carbon-13, glucose infusion, *in vivo* magnetic resonance spectroscopy, RF power deposition, specific absorption rate, high magnetic field

## Abstract

*In vivo*^13^C magnetic resonance spectroscopy (MRS) is a unique and effective tool for studying dynamic human brain metabolism and the cycling of neurotransmitters. One of the major technical challenges for *in vivo* ^13^C-MRS is the high radio frequency (RF) power necessary for heteronuclear decoupling. In the common practice of *in vivo* ^13^C-MRS, alkanyl carbons are detected in the spectra range of 10–65 ppm. The amplitude of decoupling pulses has to be significantly greater than the large one-bond ^1^H-^13^C scalar coupling (^1^J_CH_ = 125–145 Hz). Two main proton decoupling methods have been developed: broadband stochastic decoupling and coherent composite or adiabatic pulse decoupling (e.g., WALTZ); the latter is widely used because of its efficiency and superb performance under inhomogeneous B_1_ field. Because the RF power required for proton decoupling increases quadratically with field strength, *in vivo* ^13^C-MRS using coherent decoupling is often limited to lowmagnetic fields [<=4 Tesla (T)] to keep the local and averaged specific absorption rate (SAR) under the safety guidelines established by the International Electrotechnical Commission (IEC) and the US Food and Drug Administration (FDA). Alternately, carboxylic/amide carbons are coupled to protons via weak long-range ^1^H-^13^C scalar couplings, which can be decoupled using low RF power broadband stochastic decoupling. Recently, the carboxylic/amide ^13^C-MRS technique using low power random RF heteronuclear decoupling was safely applied to human brain studies at 7T. Here, we review the two major decoupling methods and the carboxylic/amide ^13^C-MRS with low power decoupling strategy. Further decreases in RF power deposition by frequency-domain windowing and time-domain random under-sampling are also discussed. Low RF power decoupling opens the possibility of performing *in vivo* ^13^C experiments of human brain at very high magnetic fields (such as 11.7T), where signal-to-noise ratio as well as spatial and temporal spectral resolution are more favorable than lower fields.

## INTRODUCTION

Since the first *in vivo* ^13^C magnetic resonance spectroscopy (MRS) study was reported in 1972 [1], ^13^C-MRS has been steadily developed into a unique and effective tool for studying dynamics of metabolism. Because most metabolically-relevant compounds contain carbon, *in vivo* ^13^C-MRS is able to detect many metabolites. One of the most common practice in ^13^C-MRS is dynamic detection, where major metabolic fluxes are measured during the infusion of ^13^C labeled substrates, such as [1-^13^C]glucose, [2-^13^C]acetate, or [3-^13^C]lactate [2–6]. A primary use of dynamic *in vivo* ^13^C-MRS is to detect alkanyl carbons of major metabolites in the spectral range of 10–65 ppm during [1-^13^C]glucose infusion. However, one of the major difficulties associated with this technique is the high radio frequency (RF) power needed to decouple the large one-bond ^1^H-^13^C scalar coupling (^1^J_CH_ = 125–145Hz) [7]. Specifically, the required RF field strength for effective decoupling has to be significantly greater than ^1^J_CH_ [7].

Because spin coupling to protons complicates the signal-to-noise ratio, interpretation and quantification of the ^13^C spectra, proton decoupling is usually considered a standard and necessary procedure in data acquisition of *in vivo* ^13^C-MRS [8, 9]. Proton decoupling is often achieved using high-power amplitude-modulated and single ^1^H frequency RF pulses to modulate the proton spins in a predetermined and cyclic fashion. Ideally, the evolution of ^13^C spins under proton decoupling appears, at data sampling intervals, to be identical to that without heteronuclear spin coupling. As such, the scalar proton couplings disappear from the ^13^C spectra. Two main proton decoupling methods have been developed: broadband stochastic (random) decoupling [10] and coherent decoupling using composite or adiabatic pulses (e.g., WALTZ) [11–14]. These two decoupling methods—described in greater detail below—differ with regard to type of modulation of the proton resonances. Most current ^13^C-MRS studies use composite pulse decoupling because it can achieve efficient broadband decoupling of protons even under conditions of severe RF field inhomogeneity generated by surface transceiver coils [11–19]. Because chemical shift dispersion is proportional to static magnetic field strength (γB_0_), the proton decoupling bandwidth and the decoupling RF field strength (γB_2_) increase linearly with γB_0_. Therefore, RF power required for proton decoupling increases quadratically with field strength. Human studies using composite pulse decoupling are traditionally limited to relevant low magnetic fields [≤4 Tesla (T)] [3, 15, 20, 21] to keep the decoupling power levels lower than the threshold of the specific absorption rate (SAR) set by the International Electrotechnical Commission (IEC) [22] and the US Food and Drug Administration (FDA) [23].

Surface or half-volume transceiver coils are usually used for proton decoupling in *in vivo* ^13^C-MRS studies to obtain necessary RF efficiency and to keep the RF power deposition within an acceptable range [16]. As a result, most ^13^C-MRS human studies are performed in the occipital lobe region. The frontal lobe is usually avoided to prevent potential damage to the poorly perfused eye by high decoupling RF power deposition [24–26]. B_1_ inhomogeneties are another problem associated with surface coils. Under an inhomogeneous B_1_ field, decoupling efficiency is degraded due to spatial differences in decoupling RF fields. Under a weak coupling field, composite decoupling sequences, such as WALTZ, do not perform well because they are designed for high amplitude and fast repetition of decoupling cycles. For the same reason—that is, keeping SAR levels within safety guidelines—current state-of-the-art volume coils are not used for *in vivo* ^13^C-MRS studies in general. A much higher RF power is required for the volume coil to generate effective RF field strength for the decoupling pulse. Because the SAR accumulates linearly with the duration of decoupling, short data sampling times have been used in a few studies with typical birdcage volume coils for whole brain decoupling [27].

Several strategies have been proposed for reducing RF power deposition by proton decoupling that would enable *in vivo* ^13^C studies to be performed on clinical scanners, even at high fields. In particular, Li and colleagues found that broadband decoupling of carboxylic/amide carbons can be achieved using random RF pulses with very low power because there is only weak long-range ^1^H-^13^C scalar couplings between carboxylic/amide carbons and protons [28–33]. They further found that turnover kinetics of glutamate (Glu) C5 from [2-^13^C]glucose infusion were identical to Glu C4 from [1-^13^C]glucose. Building on this work, they developed a strategy for *in vivo* ^13^C-MRS at high fields using [2-^13^C]glucose infusion and broadband stochastic proton decoupling with low power. The strategy of using very low RF power to decouple the carboxylic/amide carbons from protons was first demonstrated on rhesus monkey brains at 4.7T [28]. This study was the first to measure the time-course spectra of the turnover of carboxylic/amide carbons of Glu, glutamine (Gln), aspartate (Asp), and gamma aminobutyric acid (GABA). The same strategy was then applied to the occipital lobe of the human brain using a clinical 3T scanner with a home-built surface coil [29]. Low power stochastic decoupling was used and proven to be far superior to the commonly used coherent decoupling at very low decoupling power. A subsequent study demonstrated, for the first time, ^13^C-MRS of frontal lobes. The frontal lobe ^13^C-MRS study was performed at 3T using a volume coil and stochastic proton decoupling [30]. Notably, volume coils have the potential to provide ^13^C-MRS signals from multiple brain regions simultaneously. More recently, a ^13^C-MRS study of human brain at 7T was safely implemented using [2-^13^C]glucose infusion and low power broadband stochastic proton decoupling [33]. This study proved that *in vivo* ^13^C-MRS studies of human brain can be performed at high fields by detecting carboxylic/amide carbons, while nevertheless maintaining the SAR below RF safety thresholds. This method was also used in a study that simultaneously measured the brain metabolism of different ^13^C-labeled substrates in the rat brain using stochastic ^1^H decoupling at 11.7T [31].

With the advent of very high field clinical magnets, further reductions in RF power deposition become necessary. Xiang and colleagues developed a frequency-domain windowed decoupling method based on broadband stochastic proton decoupling [34]. Because protons to be decoupled in the carboxylic/amide spectral regions are clustered into two specific spectral regions, RF power applied in the empty spectral window ranging from 3.90 to 6.83 ppm between these two regions are considered wasted. According to Rayleigh’s theorem, the total power in a signal remains unchanged after Fourier Transform (FT) [35]. Xiang and colleagues applied an ideal double bandpass filter to the frequency spectrum of the stochastic decoupling pulse to eliminate frequency components outside the spectral clustered regions [34]. More than half of the RF power deposition for stochastic decoupling could be saved using this frequency-domain windowing method, which has been experimentally demonstrated for *in vivo* ^13^C-MRS of rat brain at 11.7T [34].

In contrast to the frequency-domain windowing method, Li and colleagues developed a more general time domain-based random under-sampling method aimed at decreasing decoupling power and thus the SAR for *in vivo* ^13^C-MRS [36]. A windowed decoupling scheme in the time domain was developed by taking advantage of the sparsely distributed ^13^C signals and the invariant spectral baseline. The decoupling was only required during randomly selected segments of data sampling. Instead of the complicated and computationally intensive compressed sensing algorithm [37], a simple iterative algorithm was developed to reconstruct spectra using only those randomly selected data segments. Both simulations and *in vivo* data from 7T showed that excellent spectral reconstruction could be achieved with an under-sampling rate of more than 30%, thereby corresponding to reductions in RF power deposition by proton decoupling of 30% or more [36]. It is also possible to combine the frequency-domain and time-domain windowing methods to further reduce RF power deposition due to proton decoupling.

This article briefly reviews the two major decoupling methods: broadband stochastic decoupling and coherent composite pulse decoupling. We then focus on developments of carboxylic/amide ^13^C-MRS using [2-^13^C]glucose infusion with broadband stochastic decoupling. SAR simulation analysis is introduced first, followed by a review of carboxylic/amide ^13^C-MRS studies performed at 4.7T on rhesus monkey brains, studies performed at 3T and 7T on human brains using different coils, and studies performed at 11.7T on rat brain. New and existing strategies to further reduce RF power deposition, including frequency-domain windowing and time-domain random under-sampling methods, are also discussed. Further reductions in RF power deposition due to decoupling are necessary to safely perform *in vivo* ^13^C-MRS at very high fields.

## PROTON DECOUPLING IN ^13^C-MRS

Spin coupling to one or more protons can lead to significant line-broadening and signal peak splitting into multiplets that reduce signal-to-noise ratio and spectral resolution, and therefore complicate the interpretation of ^13^C-MRS spectra. Proton decoupling is used principally as a method to simplify spectra and to improve signal-to-noise ratio and spectral resolution by removing line-broadening and spectral splitting. Proton decoupling gathers the intensity from multiplets into a singlet. In general, high-power amplitude-modulated RF signals are applied that excite all the proton nuclei and removes the splits via the scalar interactions of the proton with the ^13^C. As noted above, two major decoupling methods exist: broadband stochastic decoupling [10] and coherent decoupling using composite or adiabatic pulses [11–14].

### Random RF Decoupling

It has been well-known since the early days of NMR spectroscopy that the microscopically chaotic chemical exchange between equivalent sites of spins involved in scalar couplings lead to natural decoupling between the exchanging spins and the non-exchanging spins when the fast exchange limit is reached. Random RF decoupling is based on the similarity between the evolution of the observed heteronuclear spins when the random noise-modulated irradiation of proton spins is applied, and the chaotic chemical exchange of proton spins at the microscopically level between equivalent sites. Random RF decoupling, developed five decades ago by Ernst [10], has long been considered inferior and abandoned by the NMR community ever since the advent of modern methods that use coherent composite or adiabatic pulses for heteronuclear decoupling. The coherent heteronuclear decoupling methods are far more superior for decoupling large one-bond ^1^H-^13^C couplings. Coherent composite pulse decoupling schemes are capable of decoupling a broad bandwidth (e.g., a 10 kHz bandwidth) with a *γ* B_2_ of 1–2 kHz [12, 13]. The low *γ* B_2_ required for *in vivo* ^13^C-MRS at high magnetic field, however, makes it impossible to use any coherent decoupling sequences, composite or adiabatic. For example, weak decoupling results were obtained when applying WALTZ-4 sequence at a very low *γ* B_2_ [28].

In contrast to coherent decoupling schemes, the repetition rate of the random RF decoupling pulse sequence determines its decoupling bandwidth [10]. The entire spectral range of proton resonances that are J-coupled to the observed ^13^C spins can be decoupled. Because the long-range ^1^H-^13^C splittings are not particularly large, a moderate scaling factor can achieve perfect or nearly perfect decoupling results. We thus revived the random RF decoupling strategy of Ernst for broadband decoupling of carboxylic/amide carbons at high fields. Experimentally, a nominal *γ* B_2_ of 100Hz is sufficient to provide effective decoupling in the carboxylic and amide carbon region at 4.7T [28]. GABA C1 (182.3 ppm) and Asp C4 (178.3 ppm) were detected from the dominant and more intense signals from Glu C5 (182.0 ppm) and Gln C5 (178.5 ppm), respectively.

### Composite Pulse Decoupling

The idea of coherent decoupling can be traced to the use of a spectrally-selective RF pulse for refocusing of scalar couplings. Following excitation, the evolution of the density matrix during acquisition is given by:

(1) $$\sigma (t)=C_{x}\cos\pi J_{CH}t+2C_{y}H_{z}\sin\pi J_{CH}t;$$

where C and H are the ^13^C and proton product operators, and *J*_CH_ is the scalar coupling constant. When applying a selective ^1^H $180_{x}^{o}$ pulse after a delay *t*, the density matrix is given by:

(2) $$\sigma (t)=C_{x}\cos\pi J_{CH}t-2C_{y}H_{z}\sin\pi J_{CH}t.$$

Thus, after an additional delay *t*, the evolution due to scalar coupling is refocused:

(3) $$\sigma \;(2t)=C_{x}.$$

The decoupled ^13^C spectrum can be obtained when the data sampling intervals are set at exact multiples of 2*t*.

Coherent decoupling extends the above idea by the use of a continuous and cyclic pulse train during data sampling. The goal is to make evolution of ^13^C multiplets, under the average Hamiltonian of the irradiating proton RF field, periodically indistinguishable from that of ^13^C singlets. In order to achieve broadband proton decoupling, broadband inversion pulses must be carefully designed. The composite RF pulses were the first family of the broadband inversion pulses identified. Composite pulses use pulse blocks with different nutation angles and phases. Two typical composite pulses are $90_{x}^{o}180_{y}^{o}90_{x}^{o}$ and $90_{x}^{o}180_{-x}^{o}270_{x}^{o}$, forming the basis of MLEV [11] and WALTZ [12, 13]. Further improvements can be achieved by placing the aforementioned composite pulses into cycles. The widely usedWALTZ-4 contains four successive segments: *S_x_S_x_S*_−_*_x_S*_−_*_x_*, where *S_x_* is the original composite pulse $90_{x}^{o}180_{-x}^{o}270_{x}^{o}$, and *S*_−_*_x_* is the phase inversed version $90_{-x}^{o}180_{x}^{o}270_{-x}^{o}$. The most popular and commonly used decoupling pulse, WALTZ-16, contains four successive segments of WALTZ-4. An advantage of cycling is that the imperfection introduced by previous pulses can be compensated for later. Due to weak *γ* B_2_ field for *in vivo* ^13^C-MRS especially at high magnetic fields, the resultant long pulse duration makes phase cycling and cyclic repetition of decoupling pulses much less meaningful than in high-resolution NMR spectroscopy. As such, *in vivo* decoupling by composite (and adiabatic for the same reason) pulses at low *γ* B_2_ suffer from markedly signal loss and accompanied side bands.

## CARBOXYLIC/AMIDE ^13^C-MRS

As noted above, one of the major difficulties limiting the development of *in vivo* ^13^C-MRS was to decouple the large one-bond ^1^H-^13^C scalar couplings. Many investigators have searched for methods that focused on lowering RF power for proton decoupling, which would enable *in vivo* ^13^C-MRS studies to be performed with clinical scanners. In particular, Li and colleagues demonstrated that, since the carboxylic/amide carbons were coupled to protons via weak long-range ^1^H-^13^C scalar couplings, they can be effectively decoupled using very low RF power [28–33]. They also realized that the turnover kinetics of Glu C5 from exogenous [2-^13^C]glucose was identical to that of Glu C4 from [1-^13^C]glucose. As such, decoupling the large ^1^H-^13^C scalar couplings could be replaced by decoupling the weak couplings between carboxylic/amide carbons and protons while using exogenous [2-^13^C]glucose infusion to label the metabolites. Composite pulse decoupling sequences are widely used in proton decoupling at magnetic fields <4 Tesla because of their reasonable performance under inhomogeneous RF fields. However, at very low decoupling power strength required for high field studies, coherent decoupling schemes have only a negligible decoupling effect [28]. This is due to the reduced decoupling bandwidth associated with the extended decoupling pulse length at low field. Alternately, broadband stochastic decoupling could effectively eliminate the long-range ^1^H-^13^C coupling between protons and carboxylic/amide carbons [28, 29]. This strategy has been successfully applied to human frontal lobe and occipital lobe using volume coils at 3T and to occipital lobe using surface coils at 7T, with SAR levels well below RF safety guidelines [29, 30, 33].

The original stochastic decoupling, like WALTZ decoupling, was developed for high resolution NMR spectroscopy using homogeneous coils. For decoupling carboxylic/amide carbons *in vivo*, it shows surprisingly high performance at low decoupling field strength and high decoupling field inhomogeneity generated by surface coils. To the best of our knowledge, there has been no systematic analysis of minimal requirements of decoupling field strength either theoretically or experimentally using a homogeneous coil. Its performance as a function of decoupling field strength was empirically investigated using a surface coil at 3 Tesla, however [29].

### SAR Simulation

To prevent tissue overheating, SAR simulation analysis is usually performed before actual studies associated with potentially high RF power deposition. A commercial numerical simulation software package of the finite-difference time-domain (FDTD) method was used to simulate the B_1_ field and SAR distribution [38, 39]. The maximum local *SAR_max_* and spatially averaged *SAR_ave_* are related to the timely average transmitted RF power *RF_ave_* as:

(4) $$\mathrm{Local}\;{\mathrm{SAR}}_{\max}={\mathrm{SAR}}_{10g}\times RF_{\mathrm{ave}};$$

(5) $$\mathrm{Averaged}\;{\mathrm{SAR}}_{\mathrm{ave}}=\frac{RF_{\mathrm{ave}}}{M};$$

where M is the mass of the scanned human head and *SAR*_10_*_g_* is the maximum local SAR per 10 g tissue after normalization when the total absorbed electrical power inside the human model was 1 W at 100% duty cycle (DC). *RF_ave_* is given by a combination of *RF_DEC_*, the RF power used for decoupling, and *RF_NOE_*, the RF power for NOE:

(6) $$RF_{\mathrm{ave}}=RF_{\mathrm{DEC}}\times DC_{\mathrm{DEC}}+RF_{\mathrm{NOE}}\times DC_{\mathrm{NOE}}.$$

**FIGURE 1:**
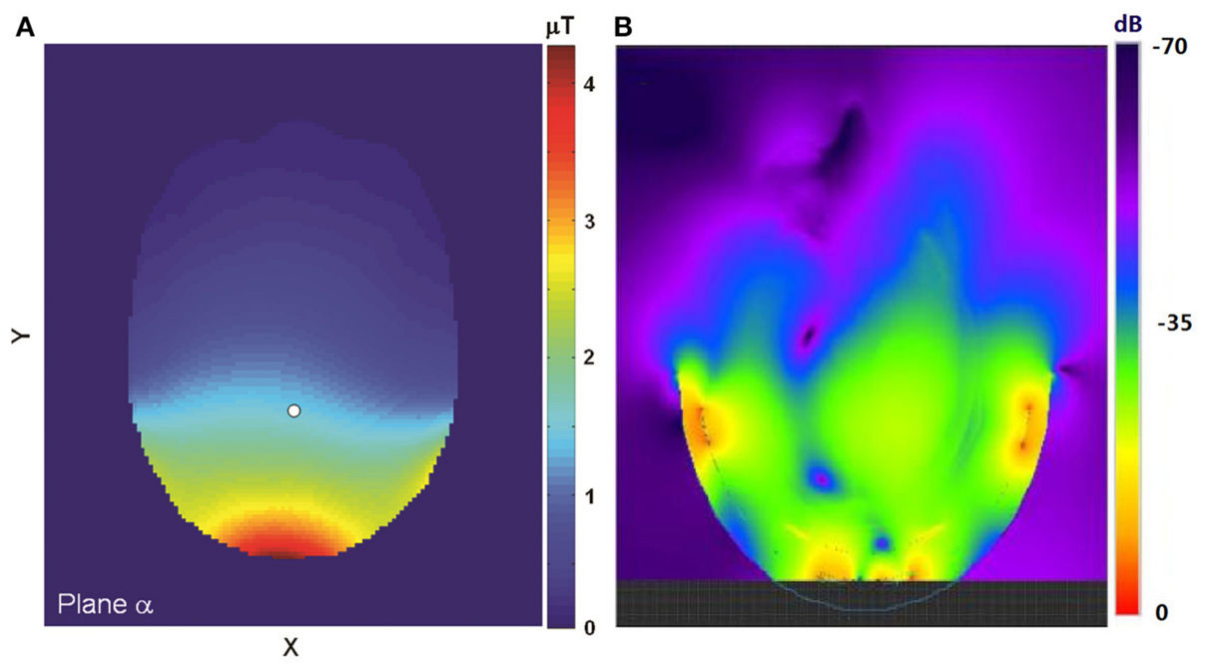
**(A)** The simulated $B_{1}^{+}$ field distribution in an axial plane at 3T. The field strength at the reference point (white dot) is 1.5 μT at the normalized condition of 1.0 W total RF absorption. Adapted from Li et al. [29]. **(B)** The simulated $B_{1}^{+}$ distribution of the proton coil in an axial plane at 7T. The upper boundary of the $B_{1}^{+}$ field display was truncated so that the field further away from the coil could be better visualized. Please note that the B_1_ field distribution shown here was extended to the RF coil, not masked by the skull. The anatomical structure of the brain overlaid on the B_1_ field map can be found in the original figure (Figure 4 in reference [33]). Adapted from Li et al. [33].

Figure 1 shows examples of the simulated $B_{1}^{+}$ field distribution of the proton coil in an axial plane at 3T (Figure 1A) and at 7T (Figure 1B). If the simulated SAR under experimental conditions is below IEC and FDA safety thresholds [22, 23], the actual experiment can be safely conducted.

### Monkey ^13^C-MRS Studies at 4.7T

**FIGURE 2:**
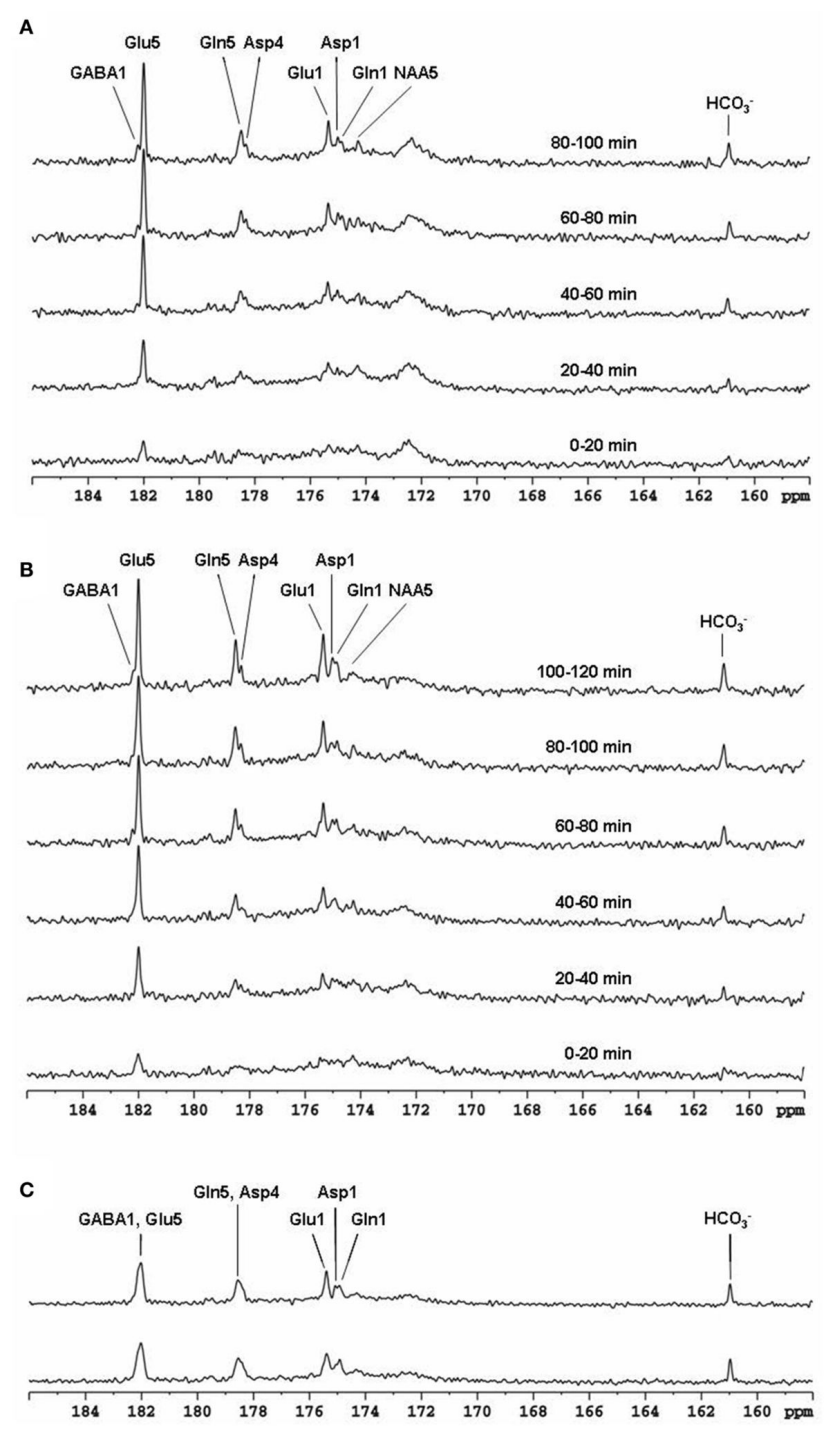
Time-course spectra of the dynamic turnover of Glu, Gln, Asp, GABA, and bicarbonate from intravenously infused [2-^13^C] Glc detected *in vivo* in the monkey brain at 4.7 Tesla using pseudo noise decoupling pulse **(A)** and vectorial noise decoupling pulse **(B)**. Spectrum acquired using WALTZ-4 decoupling (upper trace) is compared with undecoupled spectrum in **(C)**. Only a negligible decoupling effect is shown using WALTZ-4 at the low power level. Acquisition parameters for all spectra were: sweep width 10 kHz, acquisition time 204 ms, TR 2.3 s. Adapted from Li et al. [28].

The feasibility of the strategy for detecting the carboxylic/amide spectral region with low power random RF heteronuclear decoupling was first demonstrated in rhesus monkey brains at 4.7T [28]. Two decoupling schemes were tested in this study: (a) a pseudo-noise decoupling pulse with constant amplitude and randomly assigned phase (either 0° or 180°); and (b) a vectorial noise decoupling pulse with both random amplitude and phase (between 0° and 360°). The WALTZ-4 pulse with the same RF power was also evaluated as a performance benchmark. This study demonstrated that many metabolite signals, including Glu C5, Glu C1, Gln C5, Gln C1, Asp C4, Asp C1, NAA C5, and GABA C1, could be detected in the carboxylic/amide carbon spectral region (169–185 ppm) with both noise decoupling methods (Figures 2A,B). Under the same low power level, the WALTZ-4 pulse had only a negligible decoupling effect (Figure 2C). An additional advantage of this strategy is that the major metabolite resonances are not contaminated because of the absence of lipid interference. Although, fat signals pose serious spectral interference to the alkanyl spectral regions, interference due to fat signals overlapping is not an issue in the carboxylic/amide spectral region.

### Human Brain ^13^C-MRS Studies

**FIGURE 3:**
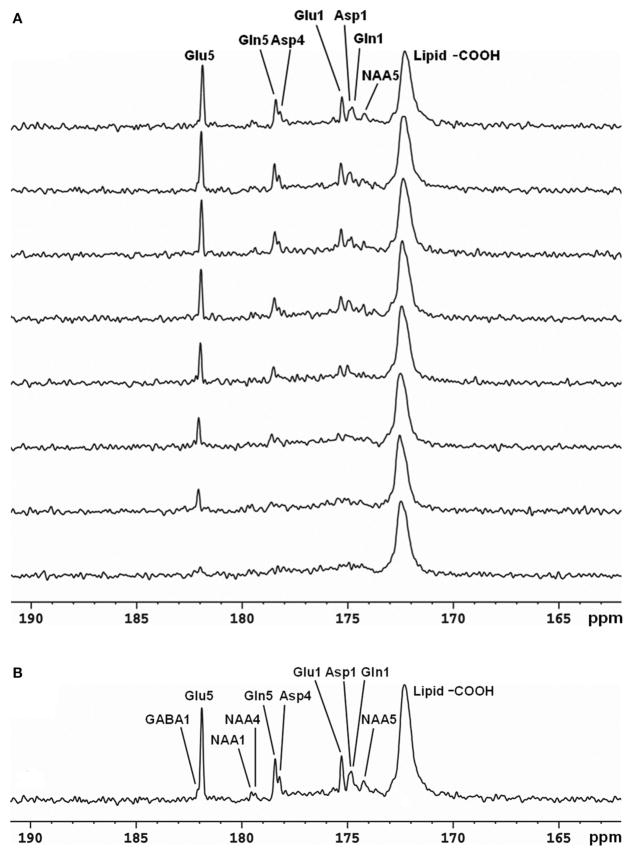
Time-course spectra of Glu, Gln, and Asp turnover detected in the occipital lobe of human brain at 3 Tesla during intravenous infusion of [2-^13^C] glucose **(A)** and summed spectrum from the last 17 min of data acquisition **(B)**. In the summed spectrum, GABA C1, NAA C4, and NAA C1 were additionally detected. Acquisition parameters were: spectral width (SW) 5 kHz, number of data points 1,024, number of scan (NS) 128, TR 4 s. Adapted from Li et al. [29].

The same strategy for detecting carboxylic/amide spectral region with low power random RF heteronuclear decoupling has been applied to human brain studies with clinical 3T [29, 30, 32] and 7T scanners [33]. The initial human study was performed on a GE 3T Excite clinical scanner [32]. A home-built RF coil consisting of a single circular ^13^C coil and a proton quadrature surface coil was used. RF decoupling and NOE were provided by a stand-alone proton decoupler (GE Healthcare). Healthy human subjects were recruited and consented to the ^13^C-MRS infusion studies. During the experiments, [2-^13^C]glucose solution was infused into an antecubital vein using an MR-compatible infusion pump. Blood samples were withdrawn periodically to measure and monitor blood glucose levels. FASTMAP automatic high-order shimming was used to optimize static magnetic field homogeneity [40]. *In vivo* ^13^C spectra were acquired using the GE product sequence (FID CSI without phase encoding) during infusion. Time course spectra of Glu, Gln, Asp, and NAA turnover from intravenously infused [2-^13^C]glucose detected in the occipital lobe are shown in Figure 3A. Glu C5 and Asp C4 were spectrally resolved. GABA C1 (182.2 ppm), NAA C4 (179.4 ppm), and NAA C1 (179.6 ppm) were also detected by summing the last 17 min of data (Figure 3B). This study also compared spectra obtained at two different stochastic decoupling power levels: 7.5 and 15 W. The spectrum acquired using 7.5Wdecoupling power showed similar results to the spectrum decoupled using 15 W; both achieved good spectral separation of Gln C5 and Asp C4.

**FIGURE 4:**
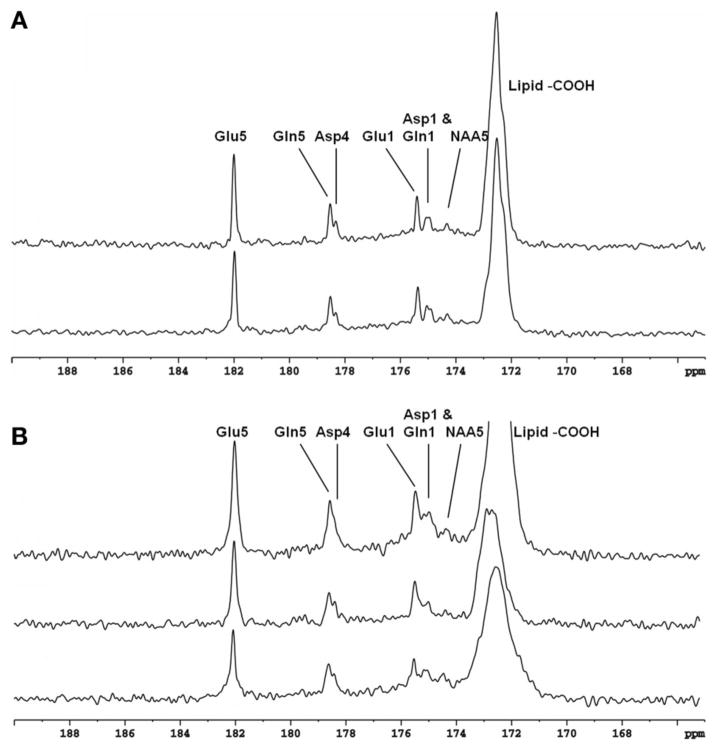
Spectra obtained from occipital lobe of two healthy volunteers **(A)** and from frontal lobe of three healthy volunteers **(B)** at 3 Tesla. A volume coil was used for stochastic proton decoupling in this study. Acquisition parameters were: number of data points 2,048, NS 384, TR 4 s. Adapted from Li et al. [30].

Half-volume transceiver coils and surface coils have been used in *in vivo* ^13^C-MRS studies for proton decoupling to keep RF power deposition within an acceptable range. However, surface coil efficiency is usually degraded under inhomogeneous B_1_ fields. In addition, and as noted above, frontal lobe regions are often avoided in ^13^C studies because the required high power for proton decoupling may cause heating of the poor perfused eyes. The strategy of using [2-^13^C]glucose infusion to detect metabolites in the carboxyl/amide region requires only very low RF power for decoupling. Using this strategy, *in vivo* ^13^C-MRS studies were conducted in human brain using volume coil decoupling at 3T [30]. The average and local SARs were evaluated using numerical simulations under experimental conditions before the actual experiments. The experimental settings in the volume coil study were the same as in the previously described surface coil study [29]. A GE 3T Excite clinical scanner (GE Healthcare, Milwaukee, WI, USA) and home-built RF coil system were used. Spectra obtained from the occipital lobe and frontal lobe of five different healthy volunteers are shown in Figure 4. In both regions, the resonances of Glu C5 (182.0 ppm), Glu C1 (175.4 ppm), Gln C5 (178.5 ppm), Gln C1 (174.9 ppm), Asp C4 (178.3 ppm), Asp C1 (175.0 ppm), and NAA C5 (174.3 ppm) were clearly detected. The occipital lobe spectra have better spectra resolution compared with that of frontal lobe spectra. This was due to the stronger B_0_ distortion caused by the nasal cavity, frontal sinus, and sphenoid sinus in the frontal lobe region.

**FIGURE 5:**
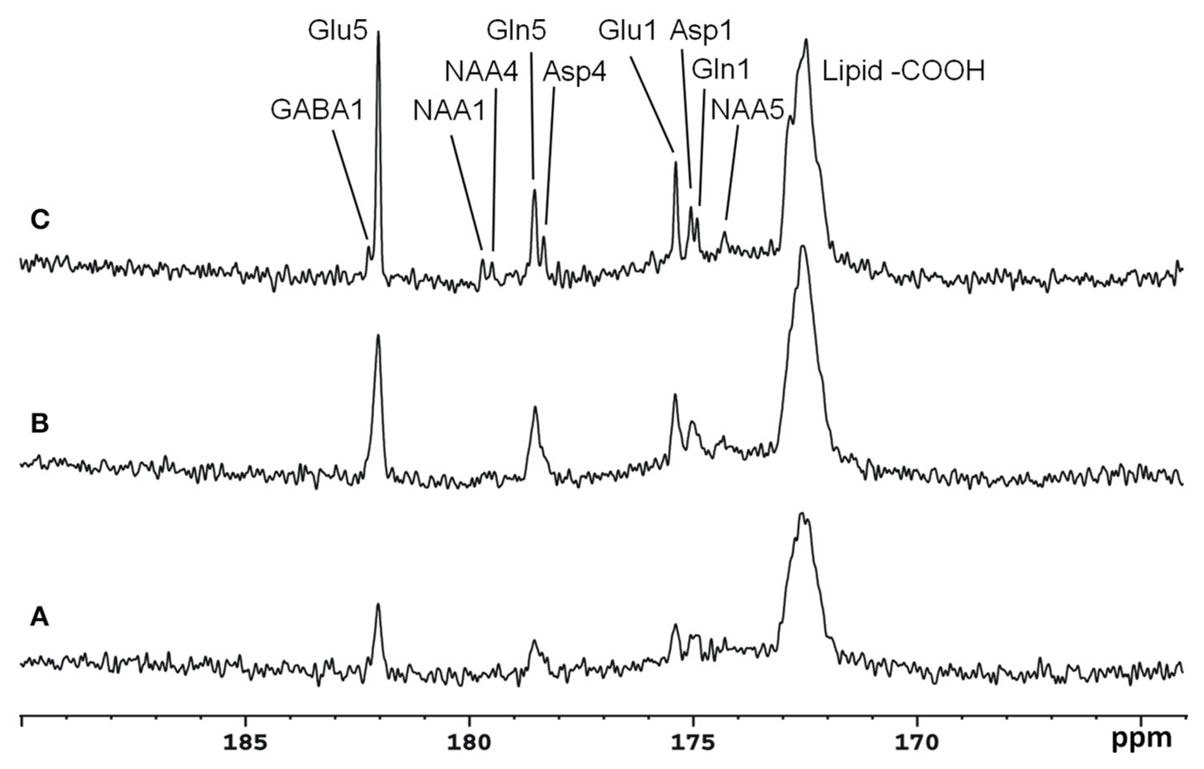
Spectra from a healthy volunteer obtained at 7 Tesla under the following conditions: **(A)** with neither NOE nor decoupling; **(B)** with NOE only; and **(C)** with both NOE and decoupling. Using the peak amplitude of Glu C5 in condition **(A)** as a reference, the peak amplitude of Glu C5 was increased on average, by a factor of 2.3 when NOE was on, and by a factor of 4.3 when both NOE and decoupling were on. Acquisition parameters for all spectra were: SW 5 kHz, number of data points 1,024, NS 104, TR 5 s. Adapted from Li et al. [33].

**FIGURE 6:**
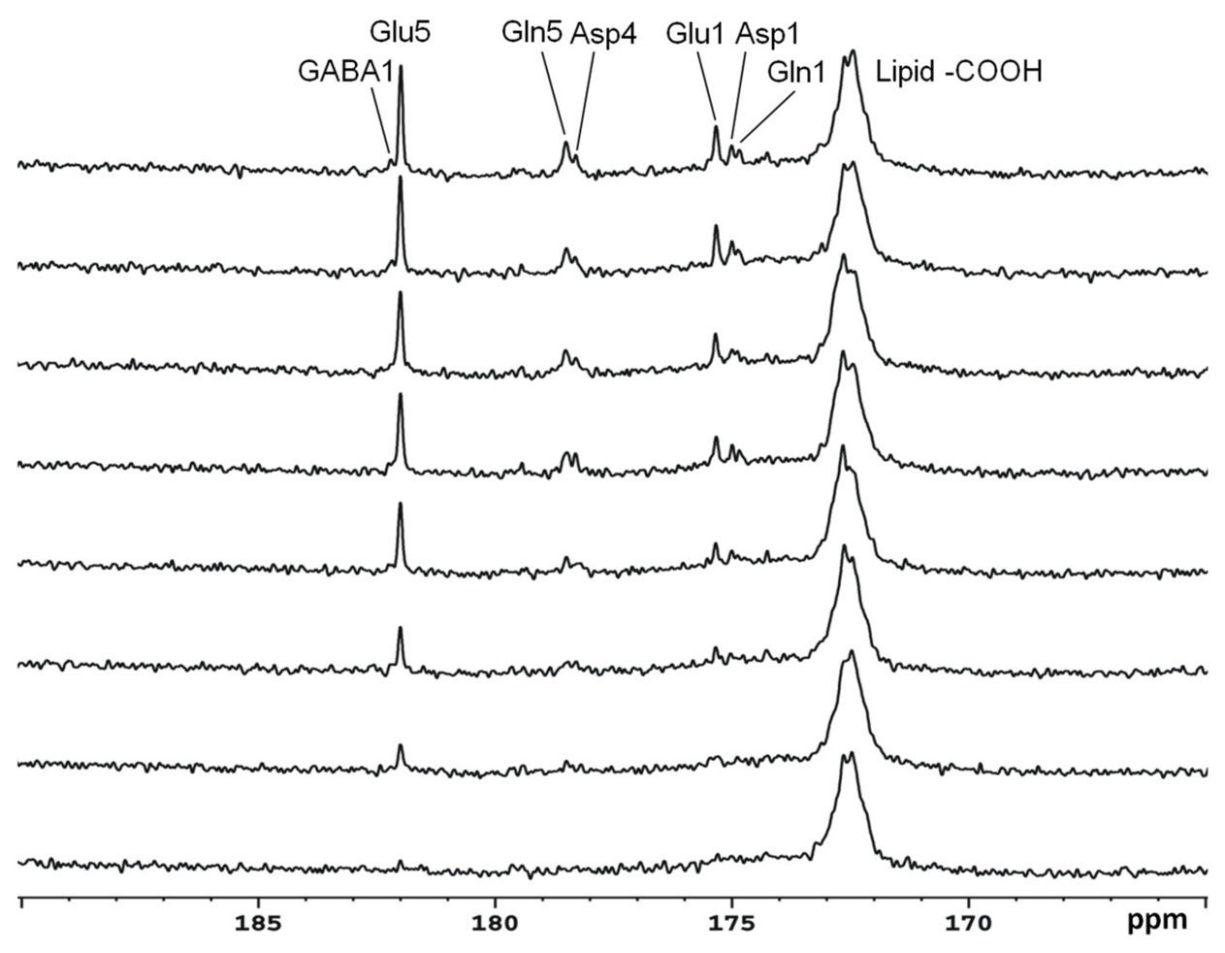
Time course spectra of Glu, Gln, and Asp turnover detected in the occipital lobe during intravenous infusion of [2-^13^C] glucose at 7 Tesla. The decoupling power was 35 W and time-averaged decoupling power was 3.6 W. Each spectrum was averaged from an 8.7 min signal with NS 104, and TR 5 s. Adapted from Li et al. [33].

**FIGURE 7:**
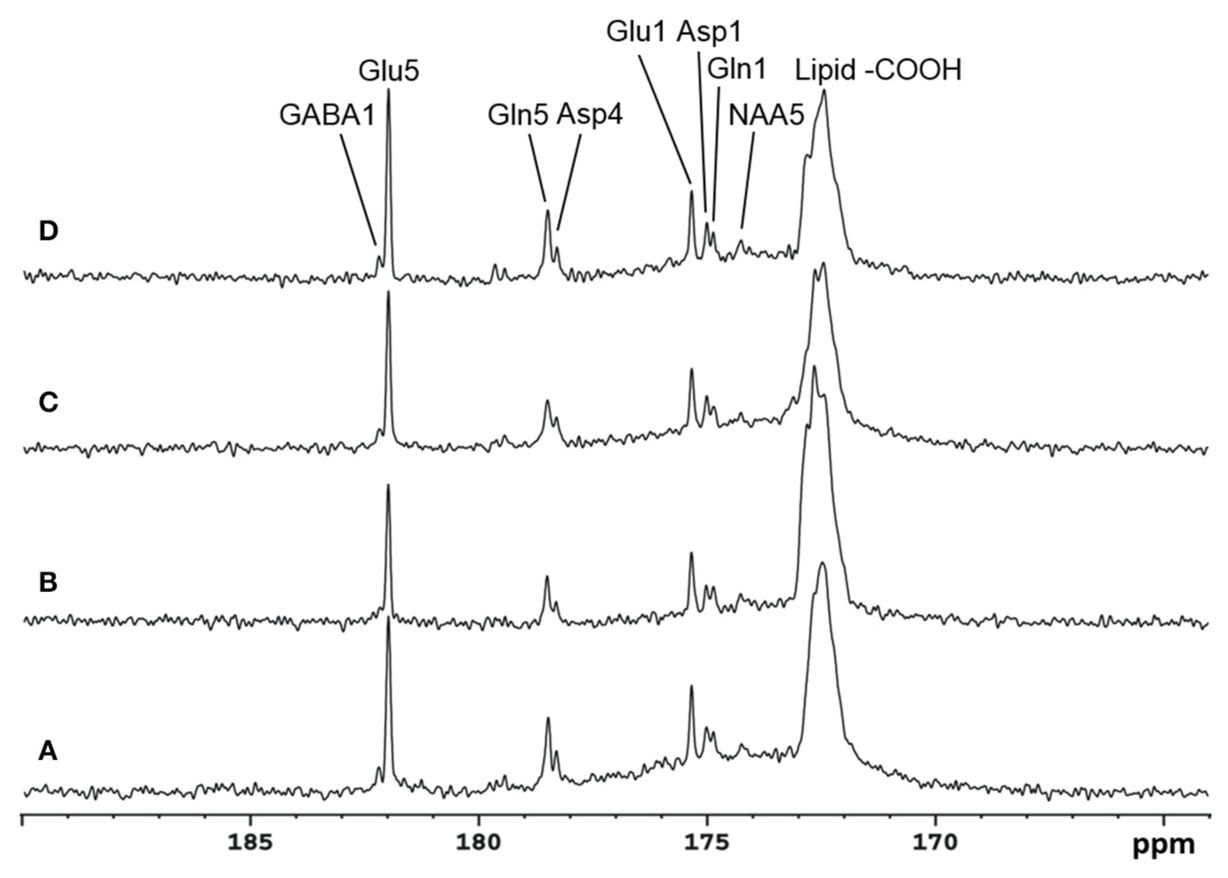
Spectra obtained from four healthy volunteers with the same NOE and decoupling power settings at 7 Tesla. The upper three traces are summed from the last 17.4 min at the end of infusion with TR 5 S, NS 208. The bottom trace is accumulated from a different subject in the last 19.6 min of data acquisition with TR 6 s, NS 192. In addition to Glu C5 and C1, Gln C5 and C1, and Asp C4 and C1, NAA C5(174.3 ppm) and GABA C1 were also clearly detected. Adapted from Li et al. [33].

Recently, the low RF power carboxylic/amide ^13^C-MRS technique was safely applied to the occipital lobe human brain studies at 7T on five healthy volunteers [33]. An in-house built RF coil assembly consisting of a circular ^13^C coil, a proton quadrature surface coil, and a slotted RF shield were used in this study. RF power of the ^13^C coil and in the proton channel were calibrated before actual data acquisition. *In vivo* ^13^C-MRS experiments were conducted on a Siemens Magneton 7T scanner during infusion of [2-^13^C]glucose. The infusion process was similar to the 3T studies. *In vivo* data were acquired using a modified Siemens FID sequence with low power broadband stochastic proton decoupling. The simulated $B_{1}^{+}$ field distribution is shown in Figure 1B. Based on the SAR calculation equations, the maximum local SAR per 10 g of tissue was 5.6 W/kg, and the average SAR was 0.9 W/kg. Both maximum and averaged SAR were well below the limits set by the IEC [22] and FDA [23]. Figure 5 shows the spectral results under three different conditions: (1) with neither NOE nor decoupling; (2) with NOE only; and (3) with decoupling and NOE. The signal peak amplitude of Glu C5 increased significantly when the combination of NOE and decoupling was used. Figure 6 shows the time course spectra of several metabolites detected in the occipital lobe with intravenous infusion of [2-^13^C] glucose. In addition to the spectrally resolved signals of Glu C5 and C1, Gln C5 and C1, Asp C4 and C1 at 3T, GABA C1 (182.2 ppm) was also observed in the time course spectra at 7T. Figure 7 shows the spectra obtained from four different subjects with the same experimental settings of NOE and decoupling power. Very similar spectra were observed among different subjects, demonstrating the reliability of the strategy with [2-^13^C] glucose infusion and low RF power stochastic decoupling. In general, GABA C1 and Glu C5 were clearly separated and distinguishable.

### Rat Brain ^13^C-MRS at 11.7T

**FIGURE 8:**
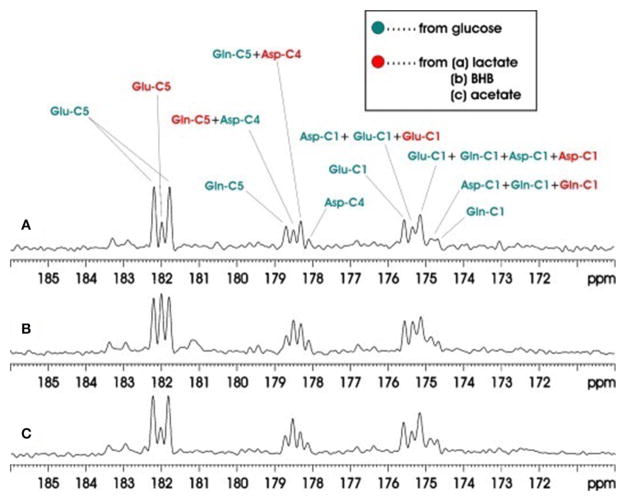
Accumulated *in vivo* ^13^C MRS spectra of intravenous co-infusions of [^13^C_6_]-d-glucose + [2-^13^C]lactate **(A)**, [^13^C_6_]-d-glucose + [1,3-^13^C_2_]BHB **(B),** and [^13^C_6_]-d-glucose + [1-^13^C]acetate **(C)**. Each spectrum was averaged over the 0–180 min infusion period from an individual rat brain. Green: resonance lines originated from [^13^C_6_]-d-glucose; red: resonance lines originated from [2-^13^C] lactate. Adapted from Xiang et al. [31].

Another study also applied carboxylic/amide ^13^C-MRS to rat brain on a Bruker microimaging spectrometer interfaced to an 11.7T 89-mm bore vertical magnet [31]. Pseudo-stochastic ^1^H decoupling was used with constant RF amplitude and randomly inverted phases. Because carboxylic and amide carbons are presented at the end of the carbon skeleton of a molecule, only singlets (doublets) ^13^C signals are showing from carboxylic/amide spectral regions when the neighboring carbon is ^12^C (^13^C). The carboxylic/amide spectrum is much simpler than the corresponding aliphatic carbon spectrum. Therefore, simultaneous detection of the metabolism contribution from different substrates can be easily determined *in vivo* from the carboxylic/amide region. Three groups of two co-infused ^13^C labeled substrates were used in this study: Group A, [^13^C_6_] glucose and [2-^13^C] lactate; Group B, [^13^C_6_]glucose and [1,3-^13^C_2_]β-hydroxybutyrate (BHB); and Group C, [^13^C_6_]glucose and [1-^13^C]acetate. Co-infusion of the different ^13^C-labeled substrates in brain demonstrated that the cerebral metabolic changes resulting from different substrates could be simultaneously measured *in vivo*. Figure 8 shows the accumulated *in vivo* ^13^C spectra of intravenous co-infusion from the three groups of ^13^C labeled substrates. [1-^13^C] acetate is the only glia-specific substrate among [^13^C_6_]glucose, [2-^13^C]lactate, [1,3-^13^C_2_] (BHB), and [1-^13^C] acetate. It labels Gln C5 in glial cells before transferring to neuronal Glu. As a result, the signal intensity of the Gln C5 singlet was much higher than that of the Glu C5 singlet (Figure 8C). Analysis of Glu C5, Gln C5, and Asp C4 signals quantified brain’s selection of its fuels for activities and functions.

## STRATEGIES FOR FURTHER REDUCTION OF DECOUPLING POWER

As described above, detecting carboxylic/amide carbons using low RF power broadband stochastic decoupling has been successfully and safely applied to human studies at 3-7T [28–33]. However, with the advent of even higher magnetic fields such as 11.7T, broadband stochastic decoupling may become problematic, given that decoupling power increases as a function of (γB_0_)^2^. Below, we review a frequency-domain windowing method and a time-domain under-sampling method developed to address this issue.

### Windowed Stochastic Proton Decoupling

In the carboxylic/amide ^13^C-MRS studies, protons that were scalarly coupled to carboxylic/amide carbons were clustered in two separate spectral regions by the resonating frequencies from alkyl protons and amide protons: 1.91 (GABA H3)-3.9 (Asp H2) ppm and 6.83 (Gln H*_z_*)-7.6 (Gln H_E_) ppm [41]. A considerable amount of RF power is wasted in the empty spectral window between these two regions. According to Rayleigh’s theorem, the total power in a signal is conserved under Fourier transform [35]. Based on this theorem, a large reduction in RF power deposition could be achieved by removing the empty spectral window spanning the 3.90–6.83 ppm region from the effective band of the stochastic decoupling sequences. To explore this possibility, Xiang and colleagues developed a windowed stochastic proton decoupling technique that multiplies a double bandpass filter to the frequency spectrum of the stochastic decoupling pulse [34]. This method skips decoupling frequency components within the empty spectral window spanning the 3.90–6.83 ppm region, thus reduced a significant amount of decoupling RF power deposition.

**FIGURE 9:**
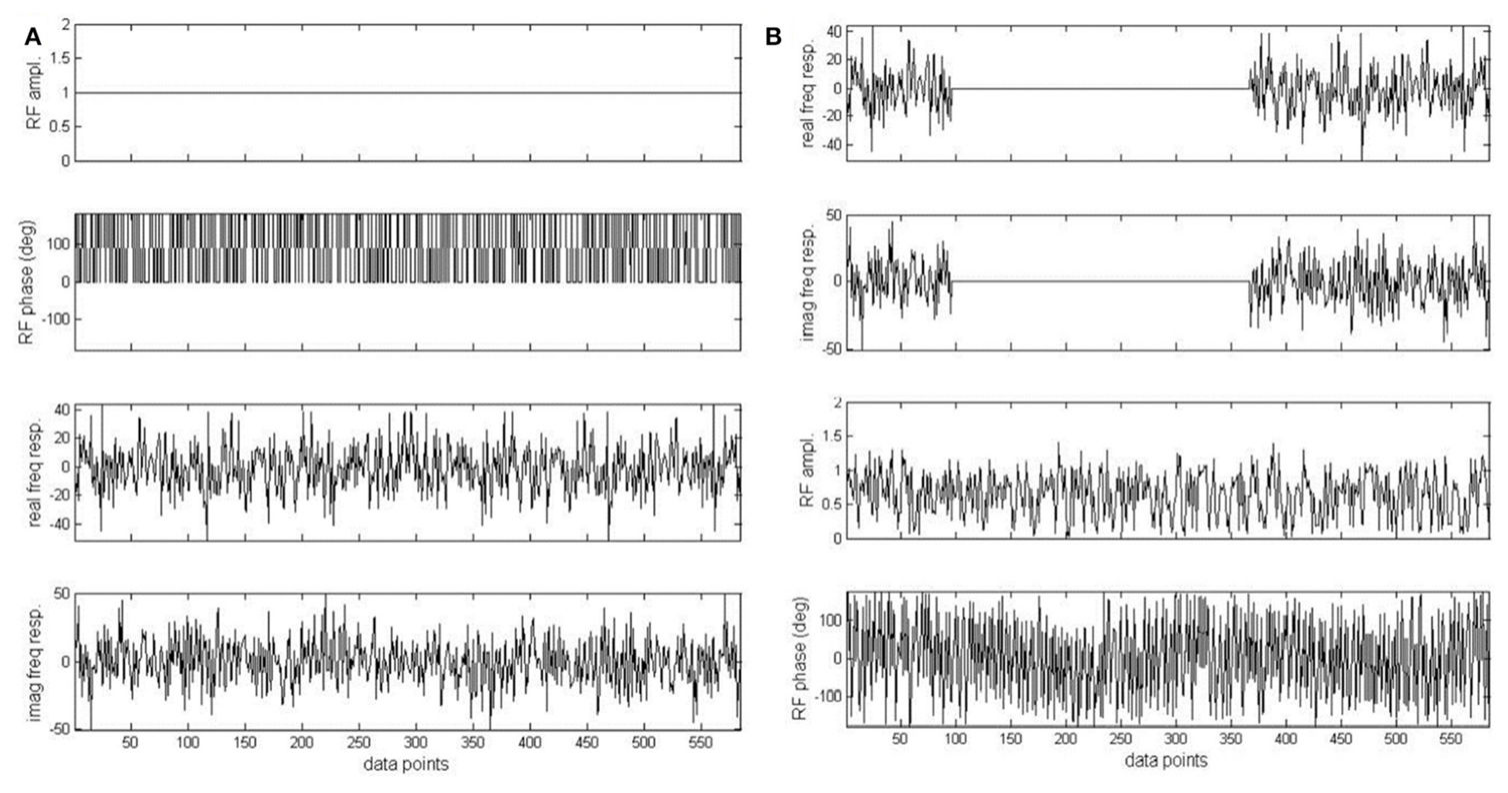
Original pseudo stochastic decoupling sequence **(A)** and the windowed version **(B)**. Phase of the original pseudo stochastic decoupling sequence was randomly chosen to be either 0 or 180°. In **(B)**, the real and imaginary component of the frequency response of **(A)** was multiplied by an ideal double bandpass filter. The two bands correspond to the 1.91–3.90 and 6.83–7.60 ppm regions with a margin of 0.1 ppm on both sides of each band. Adapted from Xiang et al. [34].

**FIGURE 10:**
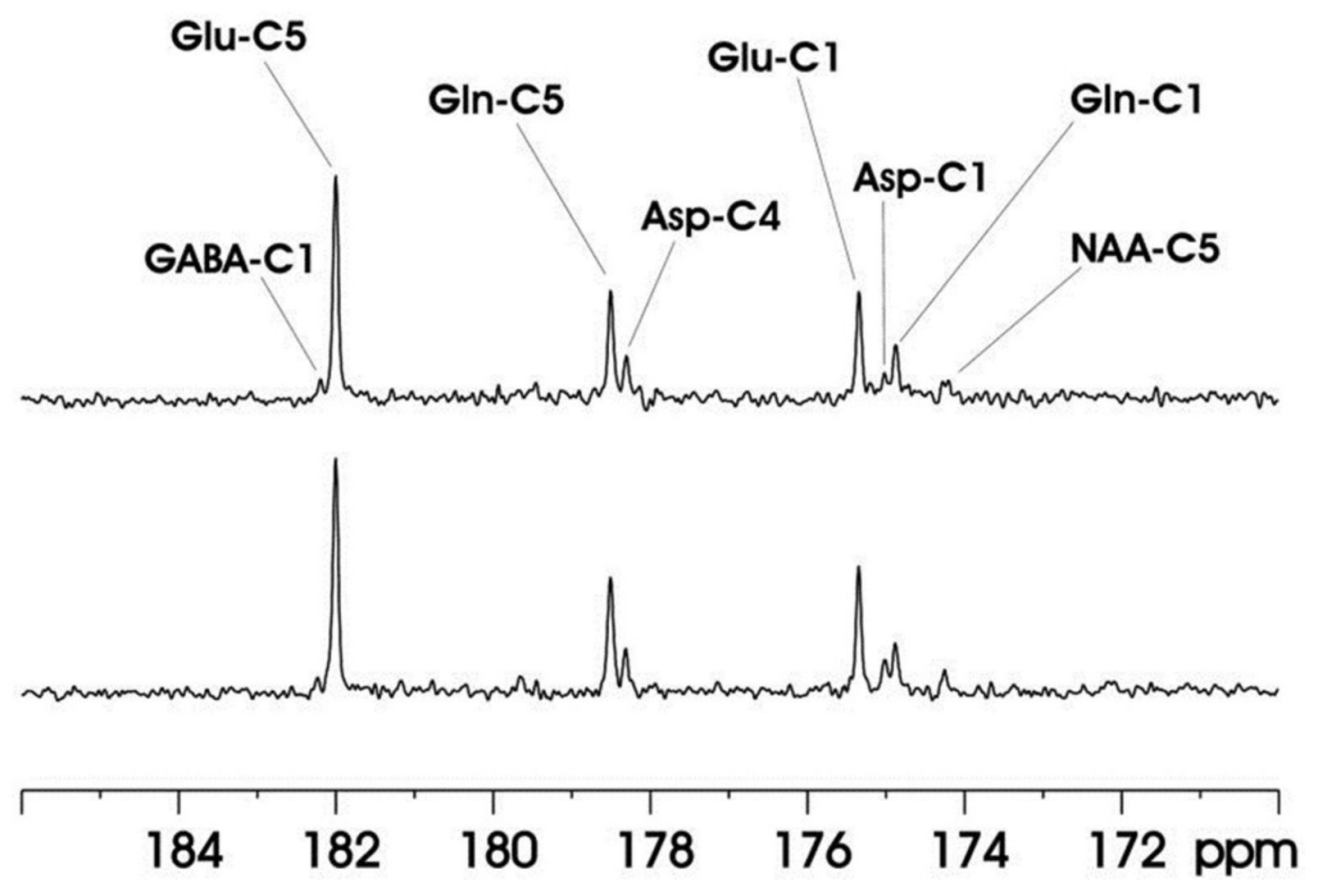
*In vivo*^13^ C MRS spectra acquired from the rat brain at 11.7 Tesla using Ernst’s stochastic decoupling sequence (top trace) and using the windowed stochastic decoupling sequence (bottom trace). In both acquisition intravenous infusion of [2,5-^13^C_2_] glucose were used. The peak RF amplitude of the windowed stochastic decoupling sequence is 707 Hz with decoupling power at 51.4% of that used in the original pseudo-stochastic decoupling sequence. Acquisition parameters were: SW 10 kHz, acquisition time 204.8 ms, TR 2 s, TE 18 ms. Adapted from Xiang et al. [34].

**FIGURE 11:**
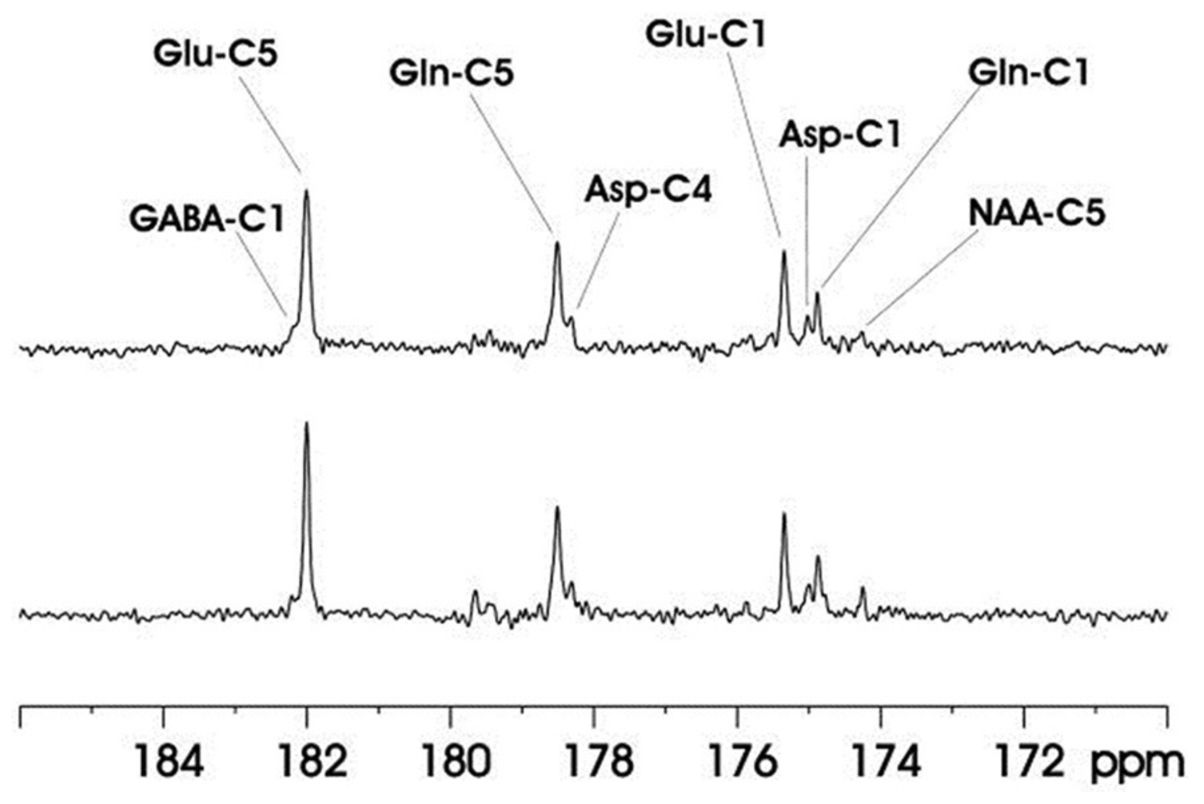
*In vivo*^13^ C MRS spectra acquired from the rat brain at 11.7 Tesla using Ernst’s stochastic decoupling sequence (top trace) and using the windowed stochastic decoupling sequence (bottom trace) at very low decoupling power. The decoupling of the original Ernst’s stochastic decoupling sequence in this study was 12.8% of that in Figure 10. Acquisition parameters were identical to Figure 10. Adapted from Xiang et al. [34].

This technique was evaluated on rat brain at 11.7T. Ernst’s pseudo-stochastic decoupling scheme was used as reference [10]. The original pseudo-stochastic decoupling sequence and the windowed version are shown in Figure 9. The double bandpass filter removes frequency components between 3.90 and 6.83 ppm. NOE was accomplished via a train of nonselective hard pulses spaced at 100 ms apart, and each with a nominal flip angle of 180°. Figure 10 compares the proton-decoupled ^13^C-MRS spectra using Ernst’s pseudo-stochastic decoupling and the windowed stochastic decoupling sequence. Decoupling power was reduced by 51.4% in windowed stochastic decoupling compared to original stochastic decoupling sequence. An essentially similar spectral decoupling effect was shown with very similar metabolite linewidth. Another comparison was also made between two decoupling methods under very low decoupling power. Figure 11 shows the proton-decoupled ^13^C-MRS spectra acquired at a decoupling power that was 12.8% of that used in Figure 10. The decoupling effect was significantly degraded using Ernst’s stochastic decoupling method, as the linewidths of Glu C5 and Gln C5 were broadened by 60.6% and 51.8%, respectively, compared to Figure 10. In contrast, much smaller linewidth increases were measured from the spectra using windowed stochastic decoupling with the same low decoupling power. This is because the RF power used for actual decoupling used in the windowed stochastic decoupling was much higher than that used in the original Ernst’s stochastic decoupling, where almost half of the decoupling power was deposited in the empty region spanning the 3.90–6.83 ppm range. As a result, the windowed stochastic decoupling method performed much better than the original stochastic decoupling method at very low RF power deposition. The further ~50% reduction in decoupling power deposition in the windowed stochastic proton decoupling method suggests that it is possible to conduct *in vivo* ^13^C-MRS studies of human brain at very high magnetic field strengths.

### Radom Time-Domain Data Sampling

The frequency-domain windowing method discussed above requires a particular signal distribution of protons that are scalarly coupled to the carbons. A more general method, based on time-domain under-sampling that does not rely on specific proton signal distribution was proposed by Li et al. [36]. In contrast to the crowded short-TE proton spectra of brain, typical *in vivo* ^13^C spectra are sparsely populated. Because sparsity of the frequency domain translates into redundant information in the time domain, a time domain under-sampling strategy was developed to further reduce the RF power deposition of proton decoupling. This new strategy used a pseudo-randomly windowed decoupling scheme in the time domain in which decoupling was only needed for randomly selected segments of data sampling. The spectra were reconstructed with an iterative algorithm using those randomly selected segments only. The random under-sampling scheme began with a fully sampled core followed by randomly sampled data segments throughout acquisition. The under-sampling rate was defined as the percentage of sampled data over the full data length.

The iterative reconstruction method began by assigning un-sampled data points *s̄* (where no decoupling power is applied) with zero values:

(7) $$x_{1}(\bar{s})=0.$$

Because of the sparsity of the spectra, a significant number of data points had zero amplitude after baseline removal. All frequency domain data points of an *in vivo* ^13^C spectrum can be classified into two different groups: (1) the signal group P, where all data points were from a metabolite peak; (2) the empty frequency group Z, where all data points had zero amplitude after baseline removal. Using this information, the iterative algorithm replaced the data in group Z by zero in the frequency domain after the forward Fourier transform (ℱ) of the under-sampled data starting from the second iteration:

(8) $$f_{n}\;(k)=\left\{ \begin{array}{c} F\;(x_{n}\;(s))\;k\in P\\ 0\;k\in Z. \end{array}\right.$$

The updated frequency domain data were transformed back into the time-domain by inverse Fourier transform (ℱ^−1^).

(9) $$x_{n}(s+\bar{s})=F^{-1}(f_{n}(k));$$

An error term *ε_n_*, defined as the absolute norm difference between the updated time domain data and the under-sampled-experimental data (30), was computed:

(10) $$\varepsilon_{n}=\mathrm{abs}|x_{n}\;(s+\bar{s})-x_{1}(s)|.$$

The iteration algorithm ended only if *ε_n_* was smaller than a manually set threshold [a very small number (<10^−6^)]. Otherwise, the algorithm proceeded by replacing the time domain data in the sampled segments with experimental data and continued to the next iteration:

(11) $$x_{n+1}(s)=x_{1}(s)$$

A maximum iteration number (>5,000) was also set to automatically end the algorithm when it failed to converge. This usually occurred when the under-sampling rate was too high.

**FIGURE 12:**
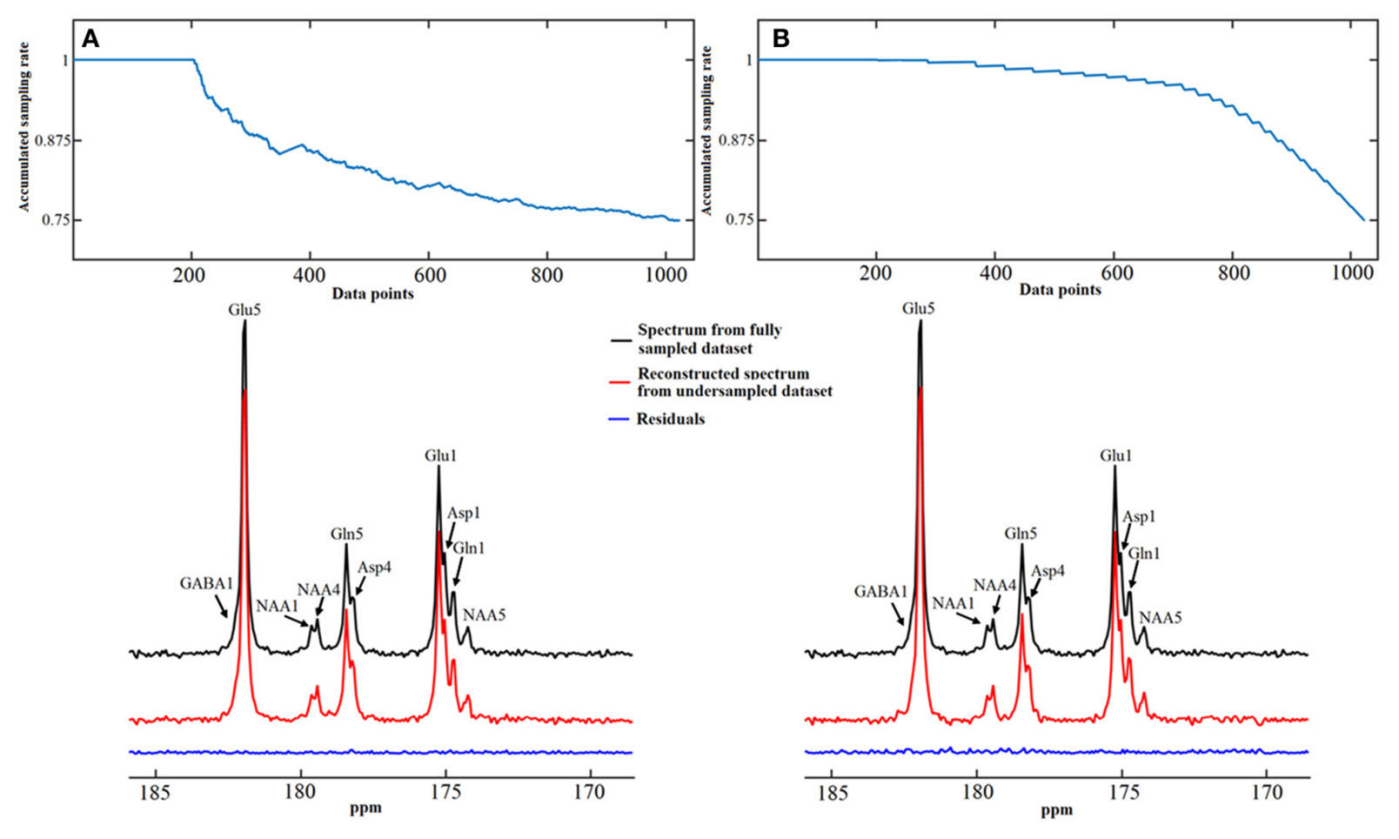
Spectra reconstruction using time-domain random under-sampling strategies. Examples of data reconstruction using the total random sampling pattern **(A)** and coherent sampling pattern with gradually decreasing under-sampling rate **(B)**. The sampling patterns from top display accumulated sampling rate over sampled data points. The last point of sampling pattern represents the overall under-sampling rate, which is 25%. Both methods began with a 20% fully sampled core. Adapted from Li et al. [36].

**FIGURE 13:**
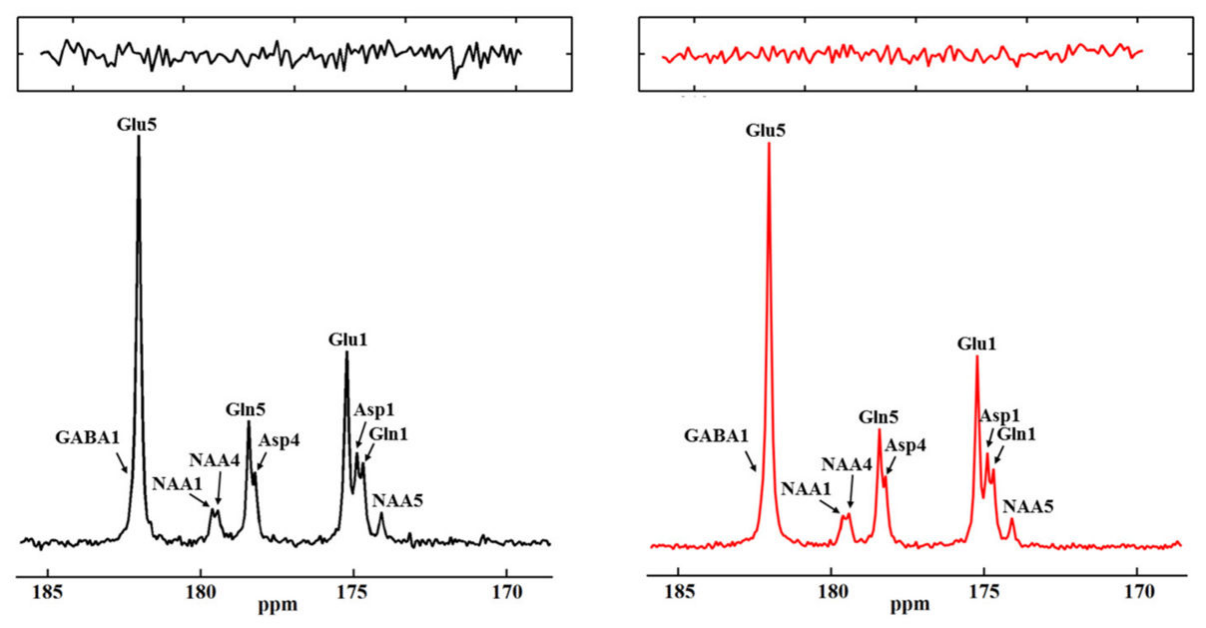
Monte Carlo simulation results from fully **(left)** and under-sampled data **(right)**. Fully sampled spectra was averaged from 70 Monte Carlo simulations and under-sampled spectra was averaged from 100 Monte Carlo simulations with a 30% under-sampling rate. The total decoupling durations are the same for the fully sampled data and under-sampled data. Zoomed in noise insets for each spectra are displayed at top. The ratio of noise variances between the fully sampled and the under-sampled spectra is 2.16:1. Adapted from Li et al. [36].

Numerical simulations were conducted to evaluate the performance of the iterative reconstruction method compared to under-sampled data obtained with different under-sampling rates and strategies. Figure 12 shows examples of spectra reconstructed using the total random sampling pattern and coherent sampling pattern with gradually decreasing under-sampling rates. In general, spectra reconstructed using the total random sampling strategy yielded fewer residuals and fewer mean signal intensity errors compared to the coherent under-sampling strategy. Therefore, total random under-sampling strategies were used in the rest of the study. Monte Carlo simulation was used to assess the SNR from the full sampling and random under-sampling for the same total number of sampled data points (therefore, the same decoupling SAR). Monte Carlo simulations demonstrated that the SNR per unit power deposition obtained via the random under-sampling strategy was significantly higher than that obtained with full sampling (Figure 13).

**FIGURE 14:**
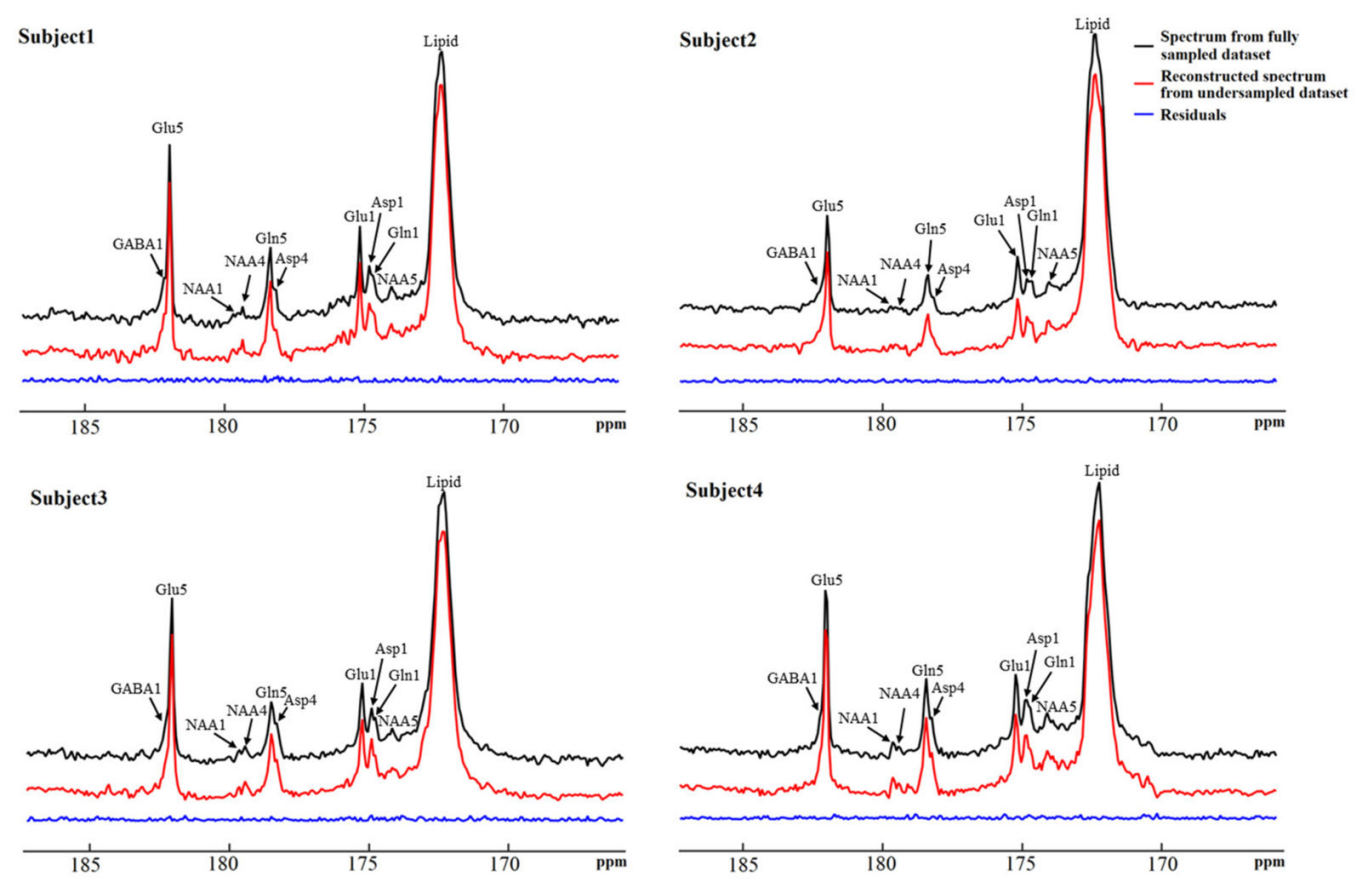
Reconstructed *in vivo* ^13^C MRS spectra from four healthy volunteers with under-sampling rate of 30%. Spectra from fully sampled data are displayed as reference. Acquisition parameters were: SW 5 kHz, data point 1,024, acquisition time 205 ms, NS 48, and TR 6s. Adapted from Li et al. [36].

**FIGURE 15:**
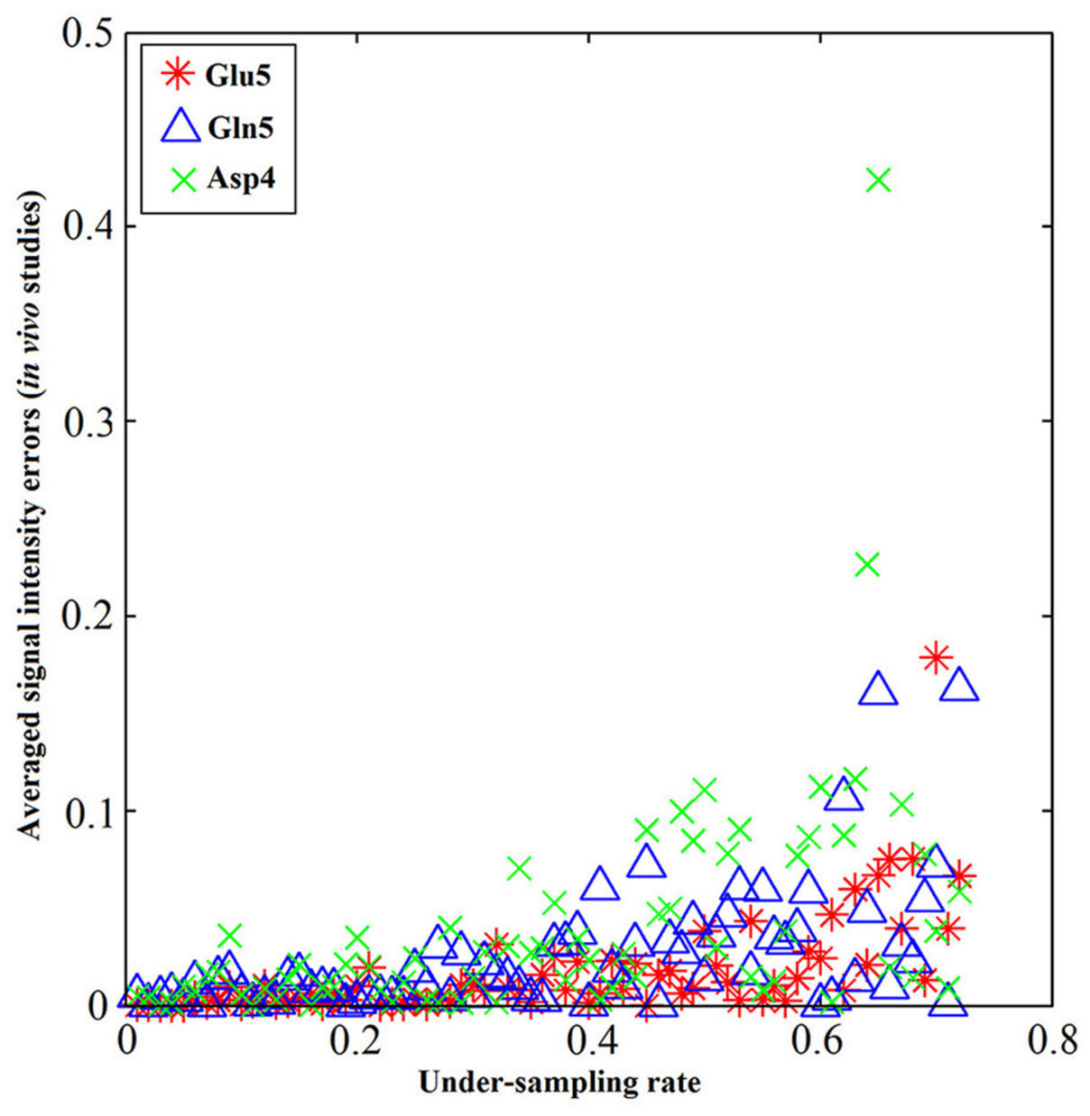
Metabolite concentrations vs. under-sampling rate from *in vivo* studies. The absolute value of signal intensity errors averaged over 4 different subjects of Glu C5 (star), Gln C5 (triangle), and Asp C4 (cross) increases as the under-sampling rate increases. The signal intensity errors for all three tested chemicals are within 5% when the under-sampling rate is less 32% in general. Similar increase trends of signal intensity errors were observed across all three chemicals as the under-sampling rate increases. Adapted from Li et al. [36].

The performance of the iterative algorithm was also evaluated on four sets of *in vivo* ^13^C human brain data acquired using a Siemens Magnetom 7T scanner [33]. A non-linear fitting algorithm developed in-house was used to generate a ^13^C baseline model from all four *in vivo* datasets [42]. The very low frequency baseline model was removed from all datasets before iterative reconstruction. Figure 14 shows the reconstructed spectra from all four *in vivo* datasets with an under-sampling rate of 30%. Visual inspection indicates only small differences between the reconstructed spectra from under-sampled data and those using the fully sampled data. Figure 15 shows the mean signal intensity error for Glu C5, Gln C5, and Asp C4 averaged from all subjects as a function of under-sampling rates. As expected, increases in the under-sampling rate led to increases in signal intensity errors. Weaker signals tended to have larger signal intensity errors.

Both simulations and *in vivo* experiments found that excellent spectral reconstruction could be achieved with a >30% under-sampling rate. This corresponded to reductions of 30% or more in RF power deposition from proton decoupling, which largely dominated the local and averaged SAR values. As no particular spectral distribution of proton signals is required for this strategy, therefore, it can be applied to alkanyl ^13^C-MRS as well. For other ^13^C regions where the signal peaks are contaminated by the broad lipid signal (e.g., muscle tissue), the usefulness of this method would be limited.

Because the SAR increases quadratically with field strength, it is always beneficial to use the lowest RF power deposition at high fields, especially for frontal lobe ^13^C studies of human subjects. The frequency-domain windowing method enhances decoupling power deposition by avoiding RF irradiation in the empty spectral region without using time-domain windowing. In contrast, the time-domain under-sampling method, in general, does not require particular spectral distributions of protons that are scalarly coupled to the carbons. Therefore, it is possible to combine the frequency-domain and time-domain windowing methods to further reduce RF power deposition for ^13^C-MRS at very high magnetic fields.

## CONCLUSION

The studies reviewed *in vivo* ^13^C-MRS using low power random heteronuclear decoupling applicable to high field studies. When combined with infusion of ^13^C labeled substrates, ^13^C-MRS becomes an excellent and unique tool for studying *in vivo* brain metabolites and investigating human diseases. A major technical challenge to developing ^13^C-MRS has been the required high RF power deposition needed to decouple large one-bond ^1^H-^13^C couplings. The evidence reviewed above demonstrates that ^13^C-MRS using [2-^13^C]glucose infusion and low power broadband stochastic proton decoupling is a viable technique for detecting metabolites at the carboxylic/amide spectral region. This technique has been safely applied to human frontal lobe and occipital lobe using volume coil at 3T and occipital lobe using surface coil at 7T, with SAR levels well below RF safety guidelines. Many metabolites, including the resonances of Glu C5, Glu C1, Gln C5, Gln C1, Asp C4, Asp C1, NAA C5, and GABA C1 can be detected and resolved. Another advantage of this technique is that the major metabolite resonances are not contaminated by lipids. Further reductions of RF power due to decoupling are accomplished by frequency-domain windowing or time-domain random under-sampling. This opens the possibility of *in vivo* ^13^C-MRS studies at very high fields.
